# Muscimol-induced inactivation of the anterior cingulate cortex does not impair trace fear conditioning in the rat

**DOI:** 10.1177/0269881120965914

**Published:** 2020-11-08

**Authors:** Marie A Pezze, Hayley J Marshall, Helen J Cassaday

**Affiliations:** School of Psychology, University of Nottingham, Nottingham, UK

**Keywords:** Reversible inactivation, muscimol, anterior cingulate cortex, trace conditioning, contextual conditioning, rat

## Abstract

Previous studies suggest that trace conditioning depends on the anterior cingulate cortex (ACC). To examine the role of ACC in trace fear conditioning further, 48 rats were surgically prepared for infusion with saline or 62.5 or 125 µg/side muscimol to inactivate ACC reversibly prior to conditioning. A noise stimulus was followed by a 1 mA footshock, with or without a 10-second trace interval between these events in a conditioned suppression procedure. The trace-conditioned groups (10 seconds) showed less test suppression than the control-conditioned groups (0 seconds). Counter to prediction, there was no effect of muscimol infusion on suppression to the noise stimulus in the 10-second trace groups.

## Introduction

We used reversible inactivation to examine further the role of the anterior cingulate cortex (ACC) in trace conditioning (Han et al., 2003; Weible et al., 2003). Micro-infusion approaches are an improvement on lesions because there is insufficient time for compensatory changes to develop, and temporary inactivation can be restricted to the conditioning stage of the procedure. We previously found little effect of dopamine D_1_ modulation of the ACC in the same fear-conditioning procedure (Pezze et al., 2016). However, dopamine D1 receptors are not the only available target to modulate ACC function (Arnsten, 2000; Robbins and Roberts, 2007). Since trace conditioning has been reported to be impaired after excitotoxic lesions in the ACC (Han et al., 2003), the very selectivity of the interventions employed by Pezze et al. (2016) may account for their lack of effect. Therefore, the GABA-_A_ receptor agonist muscimol was used to inactivate the ACC reversibly, providing a discrete intervention as spatially circumscribed as any conventional lesion but with duration of action limited to key stages of the procedure (Majchrzak and Di Scala, 2000). Thus, muscimol infusion was used to provide a better method to reproduce the effects of excitoxic lesions, with the temporal resolution afforded by micro-infusion as distinct from lesion methods. Inactivation of the ACC restricted to the conditioning stage was used to test whether ACC is necessary for the attentional and encoding processes underlying trace conditioning.

## Methods

Forty-eight experimentally naïve male Wistar rats (Charles Rivers, Edinburgh, UK; 150–200 g) were acclimatised to the laboratory and (at a minimum body weight of 250 g) implanted with bilateral guide cannulae in the ACC following the same procedure reported in Pezze et al. (2016). The experiment was run in a 3×2 design: each micro-infusion group (saline: 62.5 or 125 µg muscimol/side, injected 10 minutes prior to conditioning) was subdivided into trace and delay (10 seconds or 0 seconds) conditioning groups. Exclusions were as per a subsequent muscimol infusion study conducted on the same rats (Pezze et al., 2017): two rats were humanely killed because they did not make a good recovery postoperatively; there were three histological exclusions.

**Figure 1.**
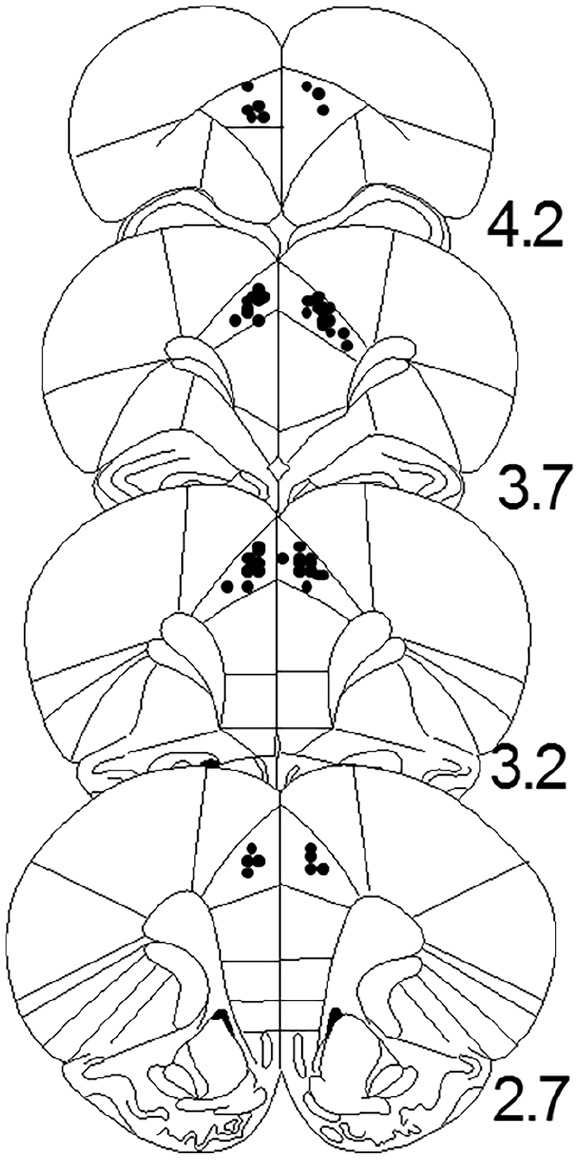
Approximate locations of infusion cannula tips in the anterior cingulate cortex. Placements are shown by black dots on coronal plates adapted from Paxinos and Watson (1998), with numbers indicating distance from bregma in millimetres.

The behavioural methods were identical to those used in Pezze et al. (2016). Following a single pair of bilateral micro-infusions, rats were exposed to two pairings of a noise conditioned stimulus (CS) followed by footshock unconditioned stimulus (US) in the trace (10 seconds) and delay (0 seconds) conditioned groups. The strength of conditioning was subsequently determined by each rat’s hesitancy to drink when returned to the conditioning box and exposed the noise CS. There was also an experimental background stimulus which was presented for the duration of the conditioning session (as per Pezze et al., 2016). Histological methods were standard (Pezze et al., 2016, 2017; Figure 1). The 3×2 factorial analyses of variance (ANOVAs) were as per Pezze et al. (2016). All procedures were carried out in accordance with the UK Animals Scientific Procedures Act 1986 (Project Licence number: PPL 40/3716).

## Results

ANOVA of baseline licking confirmed that the groups were well matched prior to conditioning (Table 1, stage A). Drinking measures taken at reshaping showed no evidence for any difference in suppression to the box cues by trace (*F*<1) or infusion on any measure (Table 1, stage B). Conditioned suppression tests to the noise CS showed a clear main effect of trace on the suppression ratio (*F*(1, 37)=16.270, *p*<0.001), first minute (*F*(1, 37)=6.043, *p*=0.019) and total licks (*F*(1, 37)=9.809, *p*=0.003) measures. However, there was no effect of infusion: across the infusion groups, rats conditioned at 10 seconds showed higher suppression ratios and drank more than the corresponding rats conditioned at 0 seconds (Table 1, stage C). There was no evidence of any difference in suppression to the light background by trace (maximum *F*(1, 37)=1.479) or infusion on any measure (Table 1, stage D).

**Table 1.** Differences in fear conditioning measures in groups conditioned at 0 and 10 seconds following infusions of saline (62.5 or 125 µg muscimol/side) into the anterior cingulate cortex at different stages of the experiment.

Stage of experiment	Measure	Saline		Muscimol 62.5 µg		Muscimol 125 µg		Statistics for main effect of infusion (*p*)	Statistics for infusion×trace interaction (*p*)
		0 seconds	10 seconds	0 seconds	10 seconds	0 seconds	10 seconds		
		*N*=5	*N*=7	*N*=8	*N*=7	*N*=8	*N*=8		
(A) Baseline licking (day 5)	Latency	11.60 (5.35)	4.14 (1.56)	3.88 (1.01)	4.57 (1.51)	8.25 (3.26)	4.00 (0.98)	0.343	0.255
	First minute licks	261 (34.7)	243 (20.0)	257 (14.8)	257 (22.3)	232 (15.5)	264 (20.6)	0.902	0.474
	Total licks	1353 (187)	1425 (59)	1400 (122)	1449 (146)	1593 (57)	1625 (147)	0.162	0.987
(B) Reshaping	Latency	48.80 (18.40)	55.71 (24.00)	65.25 (30.55)	86.29 (44.49)	176.63 (84.02)	49.13 (12.90)	0.438	0.206
	First minute licks	213 (26.2)	191 (15.1)	177 (29.7)	168 (28.4)	170 (21.4)	218 (17.0)	0.465	0.302
	Total licks	1287 (159)	1122 (109)	1252 (87)	1131 (87)	1128 (93)	1268 (143)	0.994	0.344
(C) Noise test	SR	0.03 (0.014)	0.08 (0.020)	0.02 (0.003)	0.11 (0.045)	0.03 (0.010)	0.12 (0.023)	0.723	0.570
	First minute licks	0.60 (0.2)	20.71 (9.2)	0.38 (0.2)	26.00 (22.5)	5.88 (4.9)	43.75 (20.5)	0.544	0.805
	Total licks	861 (178)	1002 (101)	610 (170)	1018 (79)	613 (187)	1257 (153)	0.669	0.292
(D) Light test	SR	0.36 (0.047)	0.41 (0.014)	0.35 (0.032)	0.38 (0.020)	0.42 (0.040)	0.43 (0.027)	0.160	0.912
	First minute licks	196 (32.6)	185 (14.2)	184 (20.5)	176 (16.2)	186 (25.8)	228 (13.5)	0.404	0.343
	Total licks	1160 (181)	1052 (153)	1311 (82)	1180 (132)	1334 (49)	1350 (137)	0.185	0.798

All data are shown as means with standard errors of the mean in parentheses. There were six experimental groups run in a 3×2 design for analysis of variance with the factors infusion (at levels saline, muscimol 62.5 µg or muscimol 125 µg) and trace condition (at levels 0 seconds or 10 seconds). The dependent variables were (A) baseline licking (day 5): latencies to drink and numbers of licks in the first minute plus in total; (B) reshaping: latencies to drink and numbers of licks in the first minute plus in total; (C) conditioned suppression to the noise CS: suppression ratios and number of licks in the first minutes plus in total; and (D) conditioned suppression to the flashing lights background stimulus: suppression ratios and number of licks in the first minute plus in total. Elevated suppression ratios and increased numbers of licks at the key noise test (C) show that conditioned suppression was reduced in the (lighter shaded) 10-second compared to the (darker shaded) 0-second trace groups. The key statistics for the main effects of trace are presented in the text. The non-significant *p-*values shown in the table are for the main effect of infusion and for the interaction term, confirming that there was no effect of infusion on trace conditioning.

SR: suppression ratio.

## Conclusion

When tested for conditioning to the CS, the trace-conditioned (10 seconds) groups showed less suppression than the control-conditioned (0 seconds) groups. Thus, there was a clear behavioural effect of the trace manipulation. Counter to prediction, there were no differences between the infusion groups. We can exclude a number of possible reasons for this negative outcome. First, rats were initially well matched in terms of their readiness to drink before conditioning: there were no pre-existing differences in baseline licking. Second, we have positive control data to show the effectiveness of the same batch of muscimol at the same concentrations, tested behaviourally (in novel object recognition procedures) using the same rats (Pezze et al., 2017). We therefore conclude that temporary inactivation in the ACC was insufficient to impair trace fear conditioning in the rat under our experimental conditions. Differences in species, extinction testing procedures and/or rostral-caudal placement of the cannulae may explain the discrepancy with previously published findings (Han et al., 2003; Weible et al., 2003). Muscimol infusions were also without effect on conditioning to the light background (contextual) stimulus. In this respect, the outcome of temporary inactivation was also different from that of dopamine D1 activation (Pezze et al., 2016).
